# Design of PCR assays to specifically detect and identify 37 *Lactobacillus* species in a single 96 well plate

**DOI:** 10.1186/s12866-020-01781-z

**Published:** 2020-04-15

**Authors:** Eiseul Kim, Seung-Min Yang, Bora Lim, Si Hong Park, Bryna Rackerby, Hae-Yeong Kim

**Affiliations:** 1 Institute of Life Sciences & Resources and Department of Food Science and Biotechnology, Kyung Hee University, Yongin, 17104 South Korea; 2 Department of Food Science and Technology, Oregon State University, Corvallis, Oregon 97331 USA

**Keywords:** *Lactobacillus*, PCR, Comparative genomics, Probiotic product, 16S rRNA gene, Species-specific primer

## Abstract

**Background:**

*Lactobacillus* species are used as probiotics and play an important role in fermented food production. However, use of 16S rRNA gene sequences as standard markers for the differentiation of *Lactobacillus* species offers a very limited scope, as several species of *Lactobacillus* share similar 16S rRNA gene sequences. In this study, we developed a rapid and accurate method based on comparative genomic analysis for the identification of 37 *Lactobacillus* species that are commonly used in probiotics and fermented foods.

**Results:**

To select species-specific sequences or genes, a total of 180 *Lactobacillus* genome sequences were compared using Python scripts. In 14 out of 37 species, species-specific sequences could not be found due to the similarity of the 16S–23S rRNA gene. Selected unique genes were obtained using comparative genomic analysis and all genes were confirmed to be specific for 52,478,804 genomes via in silico analysis; they were found not to be strain-specific, but to exist in all strains of the same species. Species-specific primer pairs were designed from the selected 16S–23S rRNA gene sequences or unique genes of species. The specificity of the species-specific primer pairs was confirmed using reference strains, and the accuracy and efficiency of the polymerase chain reaction (PCR) with the standard curve were confirmed. The PCR method developed in this study is able to accurately differentiate species that were not distinguishable using the 16S rRNA gene alone. This PCR assays were designed to detect and identify 37 *Lactobacillus* species. The developed method was then applied in the monitoring of 19 probiotics and 12 dairy products. The applied tests confirmed that the species detected in 17 products matched those indicated on their labels, whereas the remaining products contained species other than those appearing on the label.

**Conclusions:**

The method developed in this study is able to rapidly and accurately distinguish different species of *Lactobacillus*, and can be used to monitor specific *Lactobacillus* species in foods such as probiotics and dairy products.

## Background

*Lactobacillus* is a Gram-positive, non-spore-forming, rod-shaped, catalase-negative genus of bacteria that often grows best under microaerophilic conditions. *Lactobacillus* belongs to the family *Lactobacillaceae* and consists of 170 species and 17 subspecies [1]. Human and animal gastrointestinal tracts harbor a variety of *Lactobacillus* species, including *L. plantarum*, *L. rhamnosus*, *L. fermentum*, and *L*. *casei* [1], while species such as *L. gasseri*, *L. vaginalis*, *L. crispatus*, *L. iners*, and *L. jensenii* are known to exist in the vagina [2]. They have a high tolerance to acidic environments and are typically used as starter cultures for fermented foods such as kimchi, yogurt, and cheese [1]. *Bifidobacterium* and *Lactobacillus* species are among the most commercially used lactic acid bacteria (LAB) in probiotic products [3]. In particular, *L. acidophilus*, *L. casei*, *L. rhamnosus*, *L. plantarum*, and *L. paracasei* are often used in probiotic products in combination with other *Lactobacillus* species.

Probiotics are human and animal health-promoting bacteria that are generally recognized as safe (GRAS) and known to provide beneficial effects, positively affecting the intestinal microbiota, preventing urogenital infections, decreasing the effect of allergens, reducing the growth of pathogens, on the host such as gut, skin, vagina, and other sites of body [4, 5]. In recent years, the probiotic product market has expanded proportionately with an increased interest in gut health [6, 7]. Despite the widespread use of probiotic products to improve human health, there is increasing concern among consumers regarding the quality and the label claims of commercial probiotic products [3]. In terms of functionality and safety, it is very important that probiotic products contain well-documented probiotic strains that are accurately displayed on the label. However, reports have shown that the LAB species present in some commercial probiotic products do not match those represented on the label [8–10].

The traditional methods used to study microbial communities, such as morphological and physiological characteristics, protein profiling, carbohydrate fermentation patterns, and counts on selective media, are time-consuming and often produce ambiguous outcomes [11, 12]. To achieve the reliable and rapid identification of bacterial species, molecular methods such as 16S rRNA gene sequencing, metagenome sequencing, and denaturing gradient gel electrophoresis (DGGE) have been increasingly applied. 16S rRNA sequencing is commonly used for bacterial identification, including the identification of *Lactobacillus* species [13–15]. Metagenome sequencing and DGGE based on 16S rRNA gene sequences are useful analytical methods for investigating complex microbial communities without previous isolation of individual bacteria [16–18]. However, 16S rRNA gene sequences in many *Lactobacillus* species are too similar to be readily distinguished. In particular, closely related species within the *L. acidophilus* group (*L. acidophilus*, *L. gallinarum*, and *L. helveticus*), the *L. casei* group (*L. casei*, *L. paracasei*, and *L. rhamnosus*), the *L. plantarum* group (*L. plantarum*, *L. paraplantarum*, and *L. pentosus*), and the *L. sakei* group (*L. sakei*, *L. curvatus*, and *L. graminis*) are notoriously difficult to distinguish by 16S rRNA gene sequences [19, 20]. For example, the 16S rRNA gene sequence of the *L. casei* group and that of the *L. sakei* group have more than 98.7% similarity between species [19, 20].

In this study, we designed species-specific primer pairs targeting the 16S–23S rRNA gene and species-unique genes, and developed detection and identification methods for 37 *Lactobacillus* species, which are mainly used in probiotics and difficult to distinguish by conventional identification methods, using single 96 well plate of PCR assays. The developed PCR assays were applied to commercial probiotics and dairy products to distinguish *Lactobacillus* present in the product to the species level. We have also confirmed that this assay has the ability to determine the composition of *Lactobacillus* species present in a product, as well as the presence of species not stated on the label.

## Results

### Selection of species-specific sequences and primer designs

**Fig. 1:**
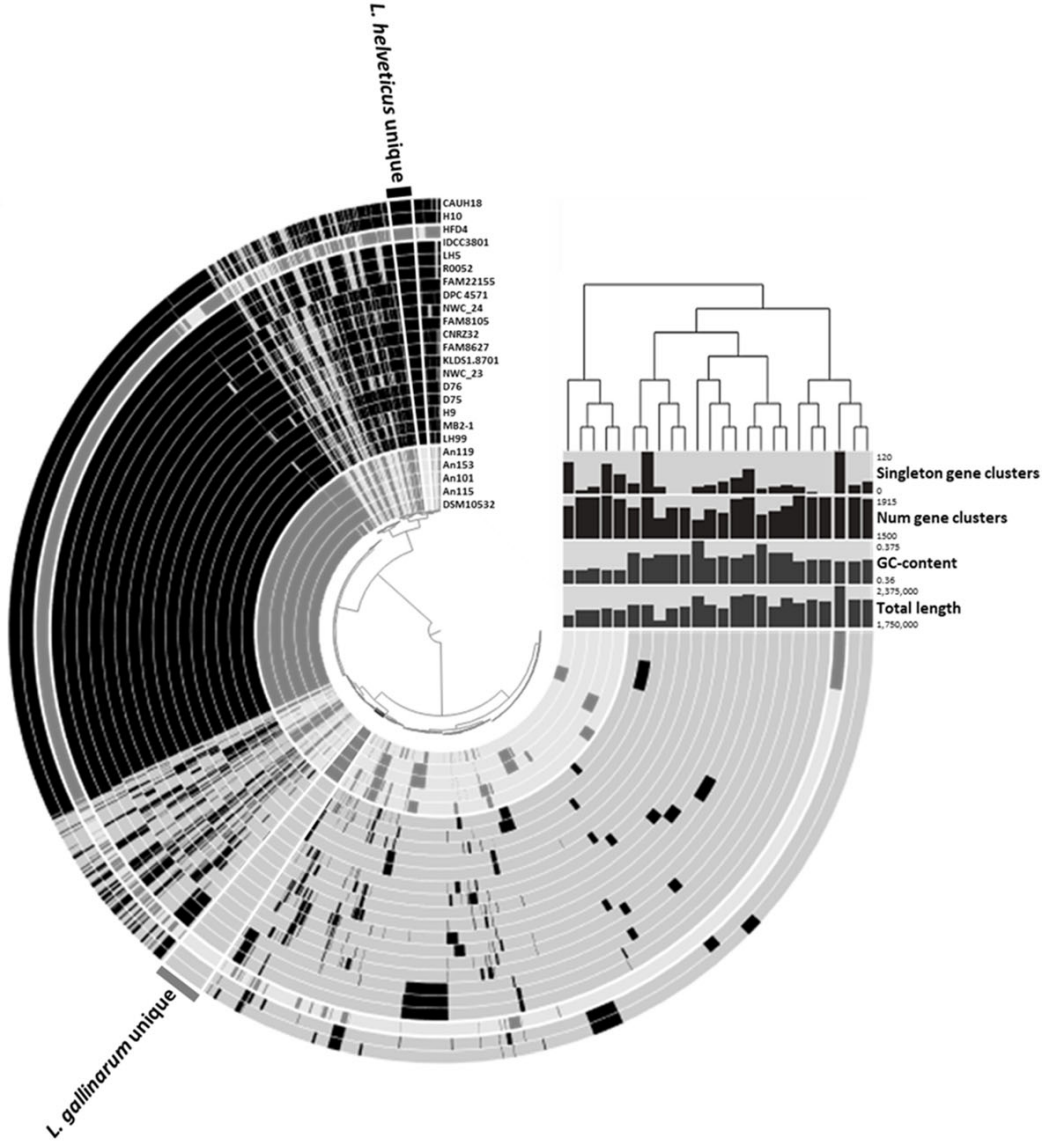
Pan-genome distribution across *Lactobacillus gallinarum* and *L. helveticus*. Each ring represents *L. gallinarum and L. helveticus* strain and each layer displays the pan-genome distribution. The gray and black rings represent the genomes of *L. gallinarum* and *L. helveticus*, respectively

The species-specific primer pairs of 37 *Lactobacillus* were designed from unique genes or the 16S–23S rRNA region (Table 1). The similarities of the 16S–23S rRNA regions among *Lactobacillus* species were verified in silico and 23 *Lactobacillus* species were distinguished with each primer pair designed in the 16S–23S region. Some *Lactobacillus* species are difficult to distinguish using the 16S–23S rRNA region alone due to the small number of single-nucleotide polymorphisms. Therefore, unique genes of 14 *Lactobacillus* species were obtained using comparative genomics (Table 2). A membrane protein was found in 4 *L. acidipiscis* genomes, but was not present in other species of *Lactobacillus*. Adenylosuccinate lyase and leucine-rich repeat protein were detected as the specific genes in *L. amylovorus* and *L. parabuchneri*, respectively. In *L. paraplantarum*, *L. plantarum*, *L. pentosus*, and *L. helveticus*, MFS (Major Facilitator Superfamily)-type transporter YcnB, LPXTG-motif cell wall anchor domain protein, GHKL domain-containing protein, and decarboxylate/amino acid:cation Na^+^/H^+^ symporter family protein were detected as the specific genes to each respective species. We also confirmed the specificity of unique genes using BLAST. The unique genes did not match any of the 52,478,804 sequences found in the NCBI database outside of the target species (Table 3). The selected unique genes confirmed to be present in the genome sequences of the reference strains with 100% identity. However, some genomes of *L. casei* contained unique genes of *L. paracasei*. The presence of unique genes in some, but not all, *L. casei* strains suggests that the genome information given for the strains is incorrect. These *L. casei* strains were found to be more similar in the 16S rRNA gene to *L. paracasei* than to the *L. casei* described in a previous study [21]. Also, one genome of *L. gallinarum* contained a unique gene of *L. helveticus*. To clarify the problem of *L. gallinarum* strain, we further performed a genomic analysis of *L. helveticus* and *L. gallinarum*. The result showed that a *L. gallinarum* strain containing a unique gene of *L. helveticus* was more similar to other strains of *L. helveticus* (Fig. 1).

**Table 1:** Information of primer pairs designed for this study

Species	Target gene	Primer name	Sequence (5′–3′)	Product size (bp)	Primer conc.^b^ (μM)
IPC^a^	16S–23S region	IPC-F	CAA CGC GAA GAA CCT TAC CAG	111	0.4
		IPC-R	CCA ACA TCT CAA CGA CAC GAG C		
*L. gasseri*	16S–23S region	Gasseri-F	TCA AGA GCT GTT AAG GCT GT	175	0.04
		Gasseri-R	CTA TCG CTT CAA GTG CTT TC		
*L. rhamnosus*	16S–23S region	Rhamnosus-F	GCC GAT CGT TGA CGT TAG TTG G	137	0.04
		Rhamnosus-R	CAG CGG TTA TGC GAT GCG AAT		
*L. brevis*	16S–23S region	Brevis-F	GGG CAA CGA AGC AAG ATC GC	260	0.08
		Brevis-R	TTC CAA TCG TGT GCA CAC CA		
*L. sakei*	16S–23S region	Sakei-F	TCG AAC GCA CTC TCG TTT AG	182	0.08
		Sakei-R	CGA AAC CAT CTT TCA ACC CT		
*L. johnsonii*	16S–23S region	Johnsonii-F	AGA GAG AAA CTC AAC TTG AAA TA	195	0.4
		Johnsonii-R	CCT TCA TTA ACC TTA ACA GTT AA		
*L. jensenii*	16S–23S region	Jensenii-F	AGT TCT TCG GAA TGG ACA TAG	148	0.4
		Jensenii-R	GCC GCC TTT TAA ACT TCT T		
*L. fermentum*	Unique gene	Fermentum-F	GAC CAG CGC ACC AAG TGA TA	129	0.08
		Fermentum-R	AGC GTA GCG TTC GTG GTA AT		
*L. plantarum*	Unique gene	Plantarum-F	GCT GGC AAT GCC ATC GTG CT	147	0.12
		Plantarum-R	TCT CAA CGG TTG CTG TAT CG		
*L. paracasei*	Unique gene	Paracasei-F	CAA TGC CGT GGT TGT TGG AA	106	0.4
		Paracasei-R	GCC AAT CAC CGC ATT AAT CG		
*L. paraplantarum*	Unique gene	Paraplantarum-F	TTA TTC AAG CCG TCG GAG TG	128	0.4
		Paraplantarum-R	TCG CTG GTG CTA ATG CAA TG		
*L. casei*	Unique gene	Casei-F	CCA CAA TCC TTG GCT GTT CT	115	0.4
		Casei-R	GCT TGA GGC GAT TGT AAT CC		
*L. curvatus*	16S–23S region	Curvatus-F	ACT CTC ATT GAA TTA GGA CGT T	132	0.4
		Curvatus-R	CCC GTG TTG GTA CTA TTT AAT		
*L. acidophilus*	16S–23S region	Acidophilus-F	CCT TTC TAA GGA AGC GAA GGA T	129	0.4
		Acidophilus-R	ACG CTT GGT ATT CCA AAT CGC		
*L. salivarius*	16S–23S region	Salivarius-F	TAC ACC GAA TGC TTG CAT TCA	138	0.08
		Salivarius-R	AGG ATC ATG CGA TCC TTA GAG A		
*L. reuteri*	16S–23S region	Reuteri-F	GAT TGA CGA TGG ATC ACC AGT	161	0.2
		Reuteri-R	CAT CCC AGA GTG ATA GCC AA		
*L. coryniformis*	16S–23S region	Coryniformis-F	CAA GTC GAA CGC ACT GAC G	165	0.4
		Coryniformis-R	ACA TTC AGG CCA TGT GGT CT		
*L. farciminis*	Unique gene	Farciminis-F	ACG AAT CCG GCA GTC AAG AA	152	0.08
		Farciminis-R	AAG AAT CGC CAA GCT CTA GG		
*L. zymae*	16S–23S region	Zymae-F	GCT AAA GCA AGC GCA CGA TT	132	0.08
		Zymae-R	TCG GCA GTG TGA CAT GGA G		
*L. pentosus*	Unique gene	Pentosus-F	GCG GTA TCG ATT CGA TTG GT	145	0.08
		Pentosus-R	TGA TGT CAA TCG CCT CTT GG		
*L. crustorum*	16S–23S region	Crustorum-F	GGA ATA GCC CAA ACC AGA G	145	0.2
		Crustorum-R	ACT GAA TGG AGT GGG TCA GA		
*L. mucosae*	16S–23S region	Mucosae-F	ACG GAC TTG ACG TTG GTT TAC	156	0.4
		Mucosae-R	GTG ATA GCC GAA ACC ACC TT		
*L. buchneri*	16S–23S region	Buchneri-F	CAA GTC GAA CGC GTC TCC AT	189	0.08
		Buchneri-R	CCG AAG CCG TCT TTT AAA CC		
*L. helveticus*	Unique gene	Helveticus-F	CTA CTT CGC AGG CGT TAA CT	132	0.08
		Helveticus-R	GTA CTT GAT GCT CGC ATA CC		
*L. amylovorus*	Unique gene	Amylovorus-F	CAA GCA CGA TTG GCA AGA TG	126	0.4
		Amylovorus-R	ATT GGA TTC CGC TTC TGT GG		
*L. heilongjiangensis*	16S–23S region	Heilongjiangensis-F	GCT TCA TGA ATC GGA TCT AA	133	0.4
		Heilongjiangensis-R	TAA ACT ACG ATC ATG TGA AAG TA		
*L. parabuchneri*	Unique gene	Parabuchneri-F	AGC GTC GTG ATT CCT GAT AC	137	0.08
		Parabuchneri-R	CGA CTC TCC GAT CGT TGT TA		
*L. acidipiscis*	Unique gene	Acidipiscis-F	AGC GGT TCG ATG GCT TAT AC	125	0.08
		Acidipiscis-R	TCC AAG TCC GAC ACC AGT CA		
*L. sanfranciscensis*	Unique gene	Sanfranciscensis-F	TGG AAC TGA TAC GCG GAT GT	130	0.08
		Sanfranciscensis-R	GGC CAA TTC CTC CAA TAA CG		
*L. ruminis*	16S–23S region	Ruminis-F	TTG CAT TCA CCG AAA GAA GC	129	0.4
		Ruminis-R	CAT AAA CAT CAT GCG GTG TTC		
*L. agilis*	16S–23S region	Agilis-F	TCG TAG CTT GCT ACA CCG ATT G	137	0.4
		Agilis-R	CAT AAT GAC CAT GCG ATC ATC A		
*L. delbrueckii*	16S–23S region	Delbrueckii-F	CAT GTG CAG ACA TGC TAT CCT T	192	0.4
		Delbrueckii-R	CTC TGA AGT GCC ATG TCT CAG T		
*L. amylophilus*	16S–23S region	Amylophilus-F	CGA GTT CTG GTT AAG AGT AGC G	174	0.4
		Amylophilus-R	CGC CAT CTT TCA AAC ATC TAT C		
*L. kunkeei*	16S–23S region	Kunkeei-F	GAA CGA GCT CTC CCA AAT TGA	161	0.4
		Kunkeei-R	GAA CCA TGC GGT TCC AAC TA		
*L. acetotolerans*	16S–23S region	Acetotolerans-F	GAT TAC CTT CGG GTA TGA AGT T	131	0.2
		Acetotolerans-R	TCA TGT GAT CTC TCC TTT TAT CC		
*L. lindneri*	Unique gene	Lindneri-F	CGG CGT TCT CGA GGA CCA TA	170	0.4
		Lindneri-R	CAT CCG GCG TCC TTC ATA GC		
*L. gallinarum*	Unique gene	Gallinarum-F	AAC TGG CGG TTA TCG TAG AC	118	0.2
		Gallinarum-R	CAC AGC AGG AAC CAT TTT AG		
*L. amylolyticus*	16S–23S region	Amylolyticus-F	TTC GGT AGT GAC GTT TCG GA	134	0.2
		Amylolyticus-R	TCA AGC AAG TGC CAT GCA G		

^a^IPC, internal positive control

^b^conc., concentration

**Table 2:** Characteristics of unique genes to each species

Species	Gene name	Accession no.
*L. sanfranciscensis*	Acetyltransferase	KRM80157.1
*L. acidipiscis*	Membrane protein	KRM26780.1
*L. fermentum*	Mannosyl-glycoprotein endo-beta-N-acetylglucosaminidase	EEI21326.1
*L. amylovorus*	Adenylosuccinate lyase	KRK41078.1
*L. pentosus*	GHKL domain-containing protein	AYJ41677.1
*L. plantarum*	LPXTG-motif cell wall anchor domain protein	EFK29584.1
*L. helveticus*	Dicarboxylate/amino acid:cation Na+/H+ symporter family protein	EEW67281.1
*L. farciminis*	DUF262 domain-containing protein	ATO45673.1
*L. parabuchneri*	Leucine-rich repeat protein	KRM47288.1
*L. paraplantarum*	MFS-type transporter YcnB	KRL48501.1
*L. gallinarum*	LacI family transcriptional regulator	KRL21687.1
*L. casei*	Putative truncated melibiose symporter	BAN74848.1
*L. paracasei*	Cation transport ATPase	ABJ68989.1
*L. lindneri*	Accessory Sec system protein Asp2	ANZ57695.1

**Table 3:** The BLASTN results of unique genes

Species	Description	Identity (%)	Target species match			Non-target species match		
			Species	No. of strains	Identify (%)	Species	No. of strains	Identity (%)
*L. sanfranciscensis*	*L. sanfranciscensis* TMW 1.1304	99	*L. sanfranciscensis*	20/20	100 ~ 98.94	–	–	–
*L. acidipiscis*	*L. acidipiscis* strain ACA-DC 1533	99.58	*L. acidipiscis*	5/5	100 ~ 99.17	–	–	–
*L. fermentum*	*L. fermentum* strain B1 28	100	*L. fermentum*	63/63	100 ~ 98.57	–	–	–
*L. amylovorus*	*L. amylovorus* DSM 20531	100	*L. amylovorus*	14/15	100 ~ 98.84	–	–	–
*L. pentosus*	*L. pentosus* strain DSM 20314	100	*L. pentosus*	22/22	100 ~ 98.35	–	–	–
*L. plantarum*	*L. plantarum* strain IDCC3501	100	*L. plantarum*	449/453	100 ~ 97.14	–	–	–
*L. helveticus*	*L. helveticus* isolate NWC_2_3	100	*L. helveticus*	56/57	100 ~ 98.70	*L. gallinarum*	1/7	99.64
*L. farciminis*	*L. farciminis* KCTC 3681	100	*L. farciminis*	7/7	100	*–*	*–*	*–*
*L. parabuchneri*	*L. parabuchneri* strain FAM21731	99.97	*L. parabuchneri*	25/25	100 ~ 96.57	*–*	*–*	*–*
*L. paraplantarum*	*L. paraplantarum* strain DSM 10667	100	*L. paraplantarum*	10/11	100 ~ 98.78	*–*	*–*	*–*
*L. gallinarum*	*L. gallinarum* DSM 10532	100	*L. gallinarum*	6/7	100 ~ 99.39	*–*	*–*	*–*
*L. casei*	*L. casei* subsp. *casei* ATCC 393	100	*L. casei*	14/25	100 ~ 96.41	*–*	*–*	*–*
*L. paracasei*	*L. paracasei* ATCC 334	100	*L. paracasei*	109/164	100 ~ 98.51	*L. casei*	3/25	98.39 ~ 98.14
*L. lindneri*	*L. lindneri* strain TMW 1.481	100	*L. lindneri*	12/12	100	–	–	–

### Specificity of designed primer pairs

To confirm whether primer pairs were species-specific for the identification of each *Lactobacillus* species, conventional PCR assays were performed with 37 *Lactobacillus* reference strains. For each of the primer pairs, the amplification product was exclusive to each target strain with a high specificity. The results of the conventional PCR assays confirmed 100% specificity for all *Lactobacillus* species.

### Specificity and accuracy of the developed PCR assays

**Fig. 2:**
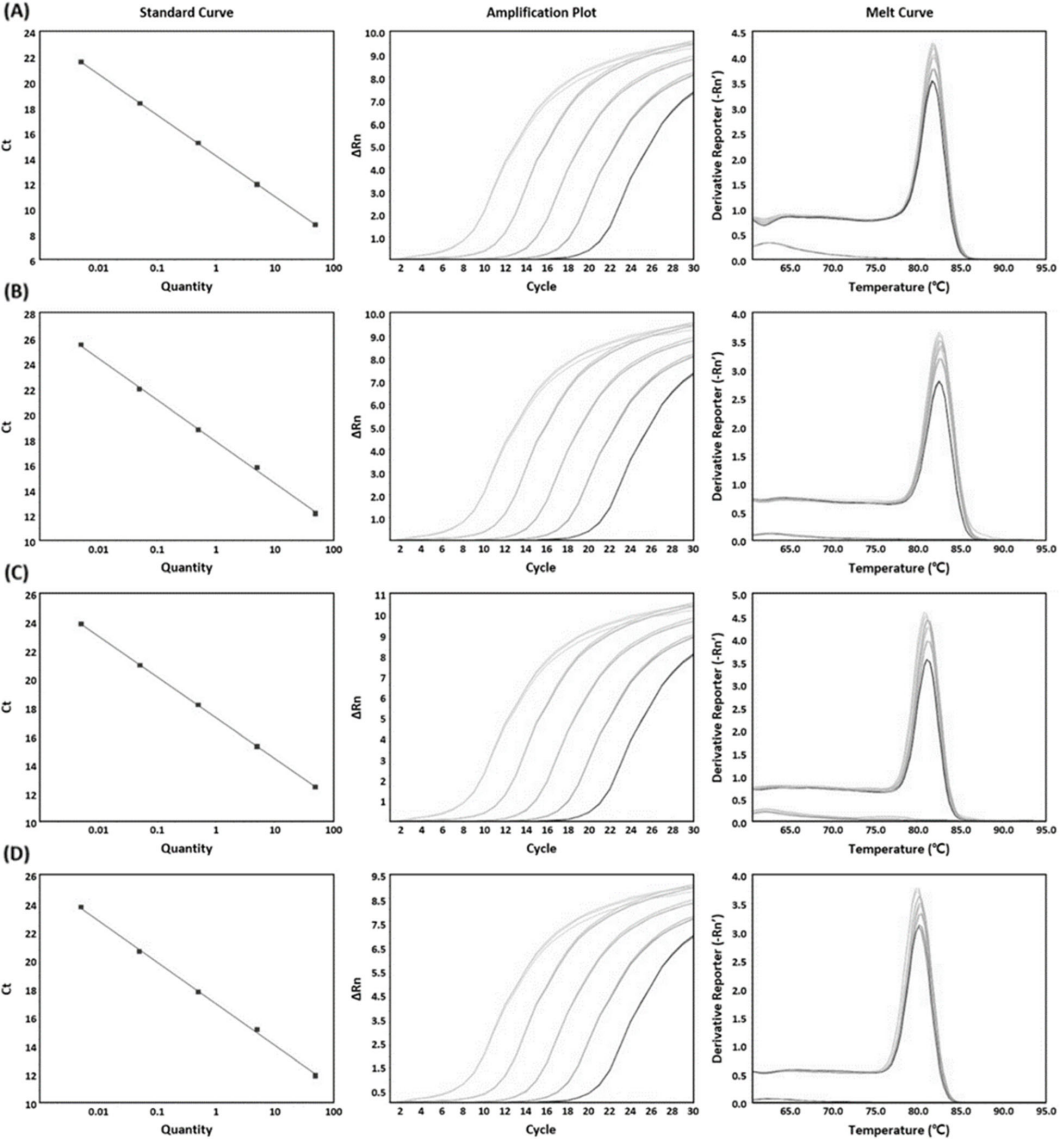
Examples of PCR standard curves, amplification curves and melting curves: **a***L. acetotolerans* standard curve between 50 and 0.005 ng (*y* = − 3.209*x* + 14.197, *R*^2^ = 1, left), amplification plot (middle) and melt curve (right); **b***L. casei* standard curve (*y* = − 3.284*x* + 17.817, *R*^2^ = 0.999, left), amplification plot (middle), melt curve (right); **c***L. parabuchneri* standard curve (*y* = − 3.207*x* + 17.19, *R*^2^ = 1, left), amplification plot (middle) and melt curve (right); and (**d**) *L. lindneri* standard curve (*y* = − 3.595*x* + 16.261, *R*^2^ = 0.982, left), amplification plot (middle) and melt curve (right)

The accuracy and efficiency of the PCR assays were validated using the template DNA of the *Lactobacillus* reference species. All primer pairs exhibited a linear relationship over the range of 0.005 to 50 ng. The slopes for the specific primer pairs of *L. acetotolerans*, *L. casei*, *L. parabuchneri*, and *L. lindneri* were − 3.209, − 3.284, − 3.207, and − 3.595, respectively, and the *R*^2^ values were 1, 0.999, 1, and 0.985, respectively (Fig. 2). The *R*^2^ and slope values of the remaining primer pairs are shown in Table 4.

**Table 4:** Slope, R^2^, and efficiency of *Lactobacillus* reference strain in the PCR assay

Species	Slope	R^2^	Efficiency (%)
*L. gasseri*	−3.214	0.999	104.701
*L. rhamnosus*	− 3.362	0.998	98.35
*L. brevis*	− 3.444	1	95.158
*L. sakei*	− 3.212	1	104.797
*L. johnsonii*	−3.214	0.999	104.701
*L. jensenii*	−3.328	0.996	99.764
*L. fermentum*	−3.56	0.995	90.955
*L. plantarum*	−3.221	0.995	104.396
*L. paracasei*	−3.305	0.98	100.694
*L. paraplantarum*	−3.256	0.998	102.822
*L. casei*	−3.284	0.999	101.612
*L. curvatus*	−3.485	0.999	93.617
*L. acidophilus*	−3.506	1	92.845
*L. salivarius*	−3.564	1	90.809
*L. reuteri*	−3.342	0.999	99.161
*L. coryniformis*	−3.217	0.989	104.578
*L. farciminis*	−3.386	0.991	97.39
*L. zymae*	−3.5	0.997	93.073
*L. pentosus*	−3.292	0.999	101.251
*L. crustorum*	−3.438	0.999	95.366
*L. mucosae*	−3.478	0.986	93.886
*L. buchneri*	−3.411	0.993	96.424
*L. helveticus*	−3.230	0.998	103.98
*L. amylovorus*	−3.582	0.993	90.167
*L. heilongjiangensis*	−3.462	1	94.458
*L. parabuchneri*	−3.207	1	105.049
*L. acidipiscis*	−3.528	0.984	92.075
*L. sanfranciscensis*	−3.229	0.999	104.034
*L. ruminis*	−3.295	1	101.153
*L. agilis*	−3.508	1	92.795
*L. delbrueckii*	−3.31	0.999	100.479
*L. amylophilus*	−3.481	0.984	93.768
*L. kunkeei*	−3.571	0.998	90.568
*L. acetotolerans*	−3.209	1	104.92
*L. lindneri*	−3.559	0.982	90.972
*L. gallinarum*	−3.346	0.999	98.989
*L. amylolyticus*	−3.552	0.996	91.209

**Fig. 3:**
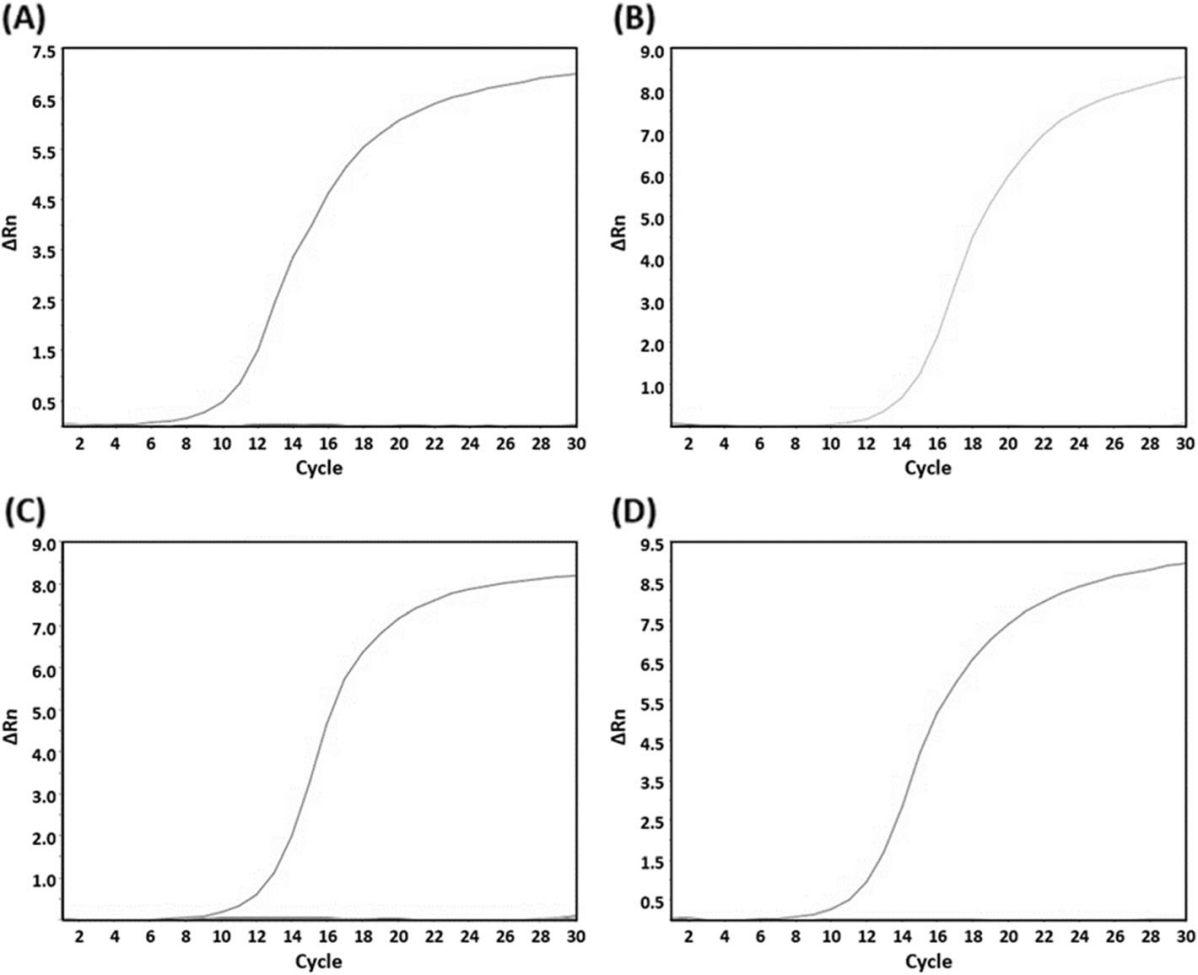
Specificities of species-specific primer pairs against 37 *Lactobacillus* species: **a** specificity of *L. acetotolerans* specific primer pair, amplification curve: *L. acetotolerans* KACC 12447; **b** specificity of *L. casei* specific primer pair, amplification curve: *L. casei* KACC 12413; **c** specificity of *L. parabuchneri* specific primer pair, amplification curve: *L. parabuchneri* KACC 12363; and (**d**) specificity of *L. lindneri* specific primer pair, amplification curve: *L. lindneri* KACC 12445

The specificities of all 37 *Lactobacillus* reference strains were evaluated for each species-specific primer pair. A non-template was used as a negative control, and the template DNA of 37 *Lactobacillus* reference stains was used as a positive control for each primer pair. All genomic DNA from *Lactobacillus* species yielded detectable amplicon signals in the well containing each primer pair, whereas none of the non-target *Lactobacillus* species generated any signals at all (Fig. 3). The C_t_ ranges were 9.0 to 15.0 for each *Lactobacillus* species (Table 5). Thus, all primer pairs were considered specific for the detection of an individual *Lactobacillus* species. To verify the accuracy of the assay, a primer pair targeting the 16S rRNA gene was used as an IPC; the amplification of the target region was observed within the C_t_ value range of 5.7 to 9.1 for all tested *Lactobacillus* species.

**Table 5:** Specificity results of the PCR assay

Primer name	Detected species	Ct value	Tm (°C)
Gasseri-F,R	*L. gasseri* KCTC 3163	5.366	83.749
Rhamnosus-F,R	*L. rhamnosus* KCTC 3237	11.258	79.329
Brevis-F,R	*L. brevis* KCTC 3498	5.762	85.319
Sakei-F,R	*L. sakei* KCTC 3603	11.139	82.441
Johnsonii-F,R	*L. johnsonii* KCTC 3801	6.450	84.193
Jensenii-F,R	*L. jensenii* KCTC 5194	6.583	81.378
Fermentum-F,R	*L. fermentum* KACC 11441	4.260	88.582
Plantarum-F,R	*L. plantarum* KACC 11451	10.715	82.027
Paracasei-F,R	*L. paracasei* KACC 12361	12.012	80.746
Paraplantarum-F,R	*L. paraplantarum* KACC 12373	10.884	82.306
Casei-F,R	*L. casei* KACC 12413	10.739	82.513
Curvatus-F,R	*L. curvatus* KACC 12415	13.832	82.686
Acidophilus-F,R	*L. acidophilus* KACC 12419	12.383	79.308
Salivarius-F,R	*L. salivarius* KCTC 3600	14.905	81.806
Reuteri-F,R	*L. reuteri* KCTC 3594	9.142	83.439
Coryniformis-F,R	*L. coryniformis* KACC 12411	13.638	84.793
Farciminis-F,R	*L. farciminis* KACC 12423	10.678	80.465
Zymae-F,R	*L. zymae* KACC 16349	7.546	82.568
Pentosus-F,R	*L. pentosus* KACC 12428	11.603	84.268
Crustorum-F,R	*L. crustorum* KACC 16344	12.467	82.012
Mucosae-F,R	*L. mucosae* KACC 12381	11.598	83.109
Buchneri-F,R	*L. buchneri* KACC 12416	11.606	82.206
Helveticus-F,R	*L. helveticus* KACC 12418	12.087	79.059
Amylovorus-F,R	*L. amylovorus* KACC 12435	11.256	82.037
Heilongjiangensis-F,R	*L. heilongjiangensis* KACC 18741	11.922	81.205
Parabuchneri-F,R	*L. parabuchneri* KACC 12363	9.377	81.604
Acidipiscis-F,R	*L. acidipiscis* KACC 12394	10.743	81.566
Sanfranciscensis-F,R	*L. sanfranciscensis* KACC 12431	10.273	79.814
Ruminis-F,R	*L. ruminis* KACC 12429	9.724	82.341
Agilis-F,R	*L. agilis* KACC 12433	11.758	82.095
Delbrueckii-F,R	*L. delbrueckii* KACC 12420	8.621	83.114
Amylophilus-F,R	*L. amylophilus* KACC 11430	10.943	82.733
Kunkeei-F,R	*L. kunkeei* KACC 19371	8.542	83.217
Acetotolerans-F,R	*L. acetotolerans* KACC 12447	11.912	82.031
Lindneri-F,R	*L. lindneri* KACC 12445	12.910	79.917
Gallinarum-F,R	*L. gallinarum* KACC 12370	10.132	78.138
Amylolyticus-F,R	*L. amylolyticus* KACC 12374	11.694	83.460

### Application of the developed PCR assays in probiotics and dairy products

The PCR assays was applied to identify *Lactobacillus* species from commercial probiotics and dairy products. A total of 31 products were evaluated using the PCR assays we have developed, and the assay results were compared with the probiotic label claims. Probiotic products were tagged as P1 to P19, whereas dairy products were designated as D1 to D12. As a result of the validation process, 17 products were confirmed to match their label claims (Table 6). However, the label claims of four products (P14, P15, P17, and P18) identified *L. helveticus* but contained *L. acidophilus*, and three products (P14, P15, and P17) contained *L. paracasei* instead of the *L. casei* indicated on the label. In one product (P16), we detected additional *Lactobacillus* species that were not listed on the label. We were also able to identify the *Lactobacillus* species from products labeled with the compound LAB. Our PCR results confirmed that these products contained either *L. acidophilus* and *L. delbrueckii* or *L. paracasei* and *L. helveticus*.

**Table 6:** Results of application test of the developed PCR assay to commercial probiotic and dairy products

Name	Country	Label claim	Detected species
P1	Korea	*L. plantarum*	*L. plantarum*
P2	USA	*L. rhamnosus*	*L. rhamnosus*
P3	Korea	*L. acidophilus*	*L. acidophilus*
P4	Korea	*L. delbrueckii*, *L. paracasei*	*L. delbrueckii*, *L. paracasei*
P5	Korea	*L. acidophilus*, *L. rhamnosus*	*L. acidophilus*, *L. rhamnosus*
P6	Korea	*L. acidophilus*, *L. rhamnosus*	*L. acidophilus*, *L. rhamnosus*
P7	Korea	*L. acidophilus*, *L. delbrueckii*	*L. acidophilus*, *L. delbrueckii*
P8	Korea	*L. acidophilus*, *L. plantarum*, *L. reuteri*	*L. acidophilus*, *L. plantarum*, *L. reuteri*
P9	Korea	*L. acidophilus*, *L. plantarum*, *L. reuteri*	*L. acidophilus*, *L. plantarum*, *L. reuteri*
P10	Korea	*L. acidophilus, L. fermentum, L. plantarum*	*L. acidophilus*, *L. fermentum*, *L. plantarum*
P11	USA	*L. acidophilus*, *L. brevis*, *L. casei*, *L. delbrueckii*, *L. paracasei, L. plantarum*, *L. salivarius*	*L. acidophilus*, *L. brevis*, *L. casei*, *L. delbrueckii*, *L. paracasei, L. plantarum*, *L. salivarius*
P12	Canada	*L. acidophilus*, *L. casei*, *L. gasseri*, *L. paracasei*, *L. plantarum, L. reuteri*, *L. rhamnosus*	*L. acidophilus*, *L. casei*, *L. gasseri*, *L. paracasei*, *L. plantarum*, *L. reuteri*, *L. rhamnosus*
P13	Korea	*L. rhamnosus*	*L. rhamnosus*
P14	Canada	*L. acidophilus*, *L. casei*, *L. rhamnosus*	*L. helveticus*, *L. paracasei*, *L. rhamnosus*
P15	Canada	*L. acidophilus*, *L. casei*, *L. rhamnosus*	*L. helveticus*, *L. paracasei*, *L. rhamnosus*
P16	Korea	*L. rhamnosus*	*L. rhamnosus*, *L. helveticus*, *L. reuteri*
P17	Canada	*L. acidophilus*, *L. casei*, *L. plantarum*, *L. rhamnosus*	*L. helveticus*, *L. paracasei*, *L. plantarum*, *L. rhamnosus*
P18	Canada	*L. acidophilus*, *L. paracasei*, *L. rhamnosus*, *L. salivarius*	*L. helveticus*, *L. paracasei*, *L. rhamnosus*, *L. salivarius*
P19	Korea	*L. delbrueckii*, *L. plantarum,* LAB mixed powder	*L. delbrueckii*, *L. plantarum*, *L. amylovorus*, *L. helveticus*, *L. paracasei*, *L. rhamnosus*
D1	Korea	*L. acidophilus*, *L. casei*	*L. acidophilus*, *L. casei*
D2	Korea	*L. delbrueckii*, *L. rhamnosus*	*L. delbrueckii*, *L. rhamnosus*
D3	Korea	*L. delbrueckii*, *L. rhamnosus*	*L. delbrueckii*, *L. rhamnosus*
D4	Korea	*L. delbrueckii*, *L. rhamnosus*	*L. delbrueckii*, *L. rhamnosus*
D5	Korea	*L. rhamnosus*, LAB	*L. rhamnosus*, *L. helveticus*, *L. paracasei*
D6	Korea	LAB, probiotic LAB	*L. acidophilus*, *L. delbrueckii*, *L. paracasei*
D7	Korea	Compound LAB	*L. acidophilus*, *L. delbrueckii*, *L. fermentum*
D8	Korea	LAB	*L. acidophilus*, *L. delbrueckii*
D9	Korea	LAB	*L. helveticus*, *L. paracasei*
D10	Korea	LAB	*L. helveticus*, *L. paracasei*
D11	Korea	LAB	*L. helveticus*, *L. paracasei*
D12	Korea	LAB	*L. helveticus*, *L. paracasei*

*LAB* lactic acid bacteria

## Discussion

A variety of methods have been used to identify LAB in foods or in the environment. The most representative method is a conventional method consisting of phenotypic and biochemical tests, which have limitations in accuracy among isolates possessing similar physiological specificities and fermentation profiles at the species level [22, 23]. To overcome these difficulties, several genotype-based methods such as DGGE and metagenome sequencing have been developed [23]. In addition, metagenome sequencing based on the 16S rRNA gene is a common approach in investigating microbial communities but is limited to distinguishing similar species [24]. Because metagenome sequencing remains a time-consuming process and requires specialized equipment and techniques, it is unsuitable for analyzing a large number of samples. To combat this, we have developed PCR assays that can rapidly and easily analyze *Lactobacillus* communities in fermented foods and potentially environmental samples.

PCR is generally considered to be a rapid, sensitive, and time-saving method for the detection of bacterial species [25–27]. The accuracy of PCR is determined by the specificity of the primer pairs used. The 16S rRNA gene is considered a marker gene for bacterial genotypic analysis and is useful for the accurate identification of bacteria [12, 28]. Studies focusing on the identification of *Lactobacillus* have mainly used PCR-based molecular analysis by primer pair targeting variable regions of the 16S rRNA gene sequences [23, 29]. However, for closely related species such as the members of the *L. casei*, *L. sakei*, *L. plantarum*, and *L. acidophilus* groups, each of which has a 16S rRNA gene similarity of more than 98% [30–32], only species-specific PCR primer pairs could sufficiently differentiate species.

To overcome the limitations of the 16S rRNA gene, we developed 37 *Lactobacillus* species-specific primer pairs based on 16S–23S rRNA gene analysis and comparative genome analysis. Species-specific primer pairs were designed to have a small amplicon size (~ 260 bp) to increase amplification efficiency and detect *Lactobacillus* species present in processed foods. The specificities of the species-specific primer pairs were confirmed using the 37 *Lactobacillus* species, and amplification was observed only in the target species DNA without any cross-reactivity. Also, it was confirmed that species such as the *L. casei* group, *L. acidophilus* group, and *L. plantarum* group, which are not distinguished by the conventional identification method, were differentiable using the species-specific primer pairs. According to the CODEX guidelines, the slope values of − 3.1 to − 3.6 are considered to indicate a high PCR efficiency. The coefficient value of determination should be at least 0.98 to be considered viable data [33]. Therefore, these results demonstrate that the developed PCR assays provides high accuracy and efficiency.

The developed PCR assays was used to assess probiotics and dairy products. Using this assays, 17 products were determined to contain the *Lactobacillus* species advertised on the label. In the remaining products, the species indicated on the labels were either replaced with or contaminated by another species. For example, *L. acidophilus* was replaced by *L. helveticus* and *L. casei* was replaced by *L. paracasei* in four probiotic products. Though these products were produced by different companies, the same strains were identified. As described above, *L. acidophilus* belongs to the same group as *L. helveticus*, and *L. casei* belongs to the same group as *L. paracasei*. The likely reason a label names species other than the one detected is misidentification [20, 34]. In one product, additional *Lactobacillus* species that were not indicated on the label were detected by PCR. These were detected at much higher C_t_ values than the *Lactobacillus* species indicated on the label, suggesting that such strains were only present in low concentrations [35]. We were also able to accurately identify the species contained in products labeled compound LAB. In all of these products, we detected *L. acidophilus* and *L. delbrueckii* or *L. helveticus* and *L. paracasei*. These results confirm that our PCR assays can detect all species of *Lactobacillus* contained in these products.

Many researchers have provided evidence that the advertised contents of commercial probiotic products containing LAB are significantly different from the actual contents [25, 34]. Lewis et al. (2016) reported that only one of the 16 commercial probiotic products corresponded exactly with the *Bifidobacterium* species claimed on the label [36]. In addition, some products are inconsistent from one lot to another. These results indicate inadequate quality control for these products.

## Conclusion

In this study, we developed specific primer pairs using comparative genomics to identify *Lactobacillus* accurately and rapidly at the species level, then applied this technology in the PCR assays that can detect and identify 37 *Lactobacillus* species in a single 96 well plate. The developed PCR assays were able to accurately discriminate species that were not distinguishable by the conventional identification method. To verify the developed PCR assays, we compared the label claims of probiotics and dairy products with the *Lactobacillus* species detected using the PCR method. The PCR assays that we have developed were successfully applied to commercial probiotic and dairy products, and showed that some products did not accurately match the *Lactobacillus* species listed on their labels. Thus, this assays will be helpful for monitoring the reliability of commercial probiotic and dairy product labels. In addition to its application in probiotic products, the assays can be applied to identify *Lactobacillus* communities in various food or environmental samples.

## Methods

### Bacterial strains and probiotic and dairy products

The *Lactobacillus* reference strains were obtained from the Korean Collection for Type Cultures (KCTC; Daejeon, South Korea; https://kctc.kribb.re.kr/) and the Korean Agricultural Culture Collection (KACC; Jeonju, South Korea; http://genebank.rda.go.kr/) (Table 7). All reference strains were cultured in Lactobacilli MRS Broth (Difco, Becton & Dickinson, Sparks, MD, USA) at 30 °C for 48 h under anaerobic conditions. The probiotic and dairy products tested in this study were obtained from various markets around the world (South Korea, United States, and Canada). The samples used in this study included 19 probiotic products (10 capsule-form pharmaceuticals and 9 powder-form food supplements) and 12 dairy products manufactured by 19 different companies. All products were labeled with bacterial species or LAB compounds.

**Table 7:** *Lactobacillus* reference strains used in this study

Species	Strain no.
*L. gasseri*	KCTC^a^ 3163
*L. rhamnosus*	KCTC 3237
*L. brevis*	KCTC 3498
*L. sakei*	KCTC 3603
*L. johnsonii*	KCTC 3801
*L. jensenii*	KCTC 5194
*L. fermentum*	KACC^b^ 11,441
*L. plantarum*	KACC 11451
*L. paracasei*	KACC 12361
*L. paraplantarum*	KACC 12373
*L. casei*	KACC 12413
*L. curvatus*	KACC 12415
*L. acidophilus*	KACC 12419
*L. salivarius*	KCTC 3600
*L. reuteri*	KCTC 3594
*L. coryniformis*	KACC 12411
*L. farciminis*	KACC 12423
*L. zymae*	KACC 16349
*L. pentosus*	KACC 12428
*L. crustorum*	KACC 16344
*L. mucosae*	KACC 12381
*L. buchneri*	KACC 12416
*L. helveticus*	KACC 12418
*L. amylovorus*	KACC 12435
*L. heilongjiangensis*	KACC 18741
*L. parabuchneri*	KACC 12363
*L. acidipiscis*	KACC 12394
*L. sanfranciscensis*	KACC 12431
*L. ruminis*	KACC 12429
*L. agilis*	KACC 12433
*L. delbrueckii*	KACC 12420
*L. amylophilus*	KACC 11430
*L. kunkeei*	KACC 19371
*L. acetotolerans*	KACC 12447
*L. lindneri*	KACC 12445
*L. gallinarum*	KACC 12370
*L. amylolyticus*	KACC 12374

^a^*KCTC* Korean Collection for Type Cultures

^b^*KACC* Korean Agricultural Culture Collection

### DNA extraction

All *Lactobacillus* reference strains were grown in MRS broth at 30 °C for 48 h under anaerobic conditions. The cultured cells were harvested by centrifugation at 13,600×*g* for 5 min, after which the supernatant was removed. Genomic DNA was extracted using a bacterial genomic DNA extraction kit (Intron Biotechnology, Seongnam, South Korea) according to the manufacturer’s instructions. Total genomic DNA from the probiotic and dairy products was extracted using a DNeasy® Blood & Tissue Kit (Qiagen, Hilden, Germany) according to the method described in a previous study [37]. DNA concentration and purity were determined by absorbance using a MaestroNano® spectrophotometer (Maestrogen, Las Vegas, NV, USA).

### Identification of *Lactobacillus* species-specific regions and primer designs

In total, 180 genome sequences, which contain 37 *Lactobacillus* species, were obtained from the National Center for Biotechnology Information (NCBI; ftp://ftp.ncbi.nlm.nih.gov/genomes/) database (Additional file 1: Table S1). The 16S–23S rRNA regions, including the intergenic spacer regions, of 180 strains were extracted from the *Lactobacillus* genomes using a script written in the Python language, and the extracted regions were aligned using the Geneious program ver. 11.1.2 (Biomatters Limited, Auckland, New Zealand). According to the alignment results, primer pairs were designed on the basis of species-specific sequences in the 16S–23S rRNA gene. Some *Lactobacillus* species are difficult to distinguish at the species level because of the high degree of similarity in their 16S–23S rRNA gene sequences. For these species, we have developed species-specific primer pairs from unique genes that exist only in the target species obtained through comparative genomic analysis.

The genome sequences of target species were blasted against the genome of target species using the UBLAST function of USEARCH program ver. 9.0 [38], with 80% cutoff identity to obtain genes with high similarity [39]. The genes that showed a significant match with the genomes of all target species were considered as core genes of target species. Those genes were then blasted against all of the *Lactobacillus* genomes except the target species using the UBLAST function of USEARCH program with default parameter settings of 50% cutoff identity [38]. Genes that found no match to all genomes of the non-target species were identified as potential unique genes. The identified potential unique genes were verified using the Basic Local Alignment Search Tool (BLAST) for 52,478,804 sequences including *Lactobacillus* genomes. Also, it was confirmed whether the unique genes exist in the genome sequences of reference strains using USEARCH program. The genes were confirmed to be unique genes in the species level and found all in the target species used in this study. The species-specific primer pairs were designed based on these genes. To verify the presence of genomic DNA from *Lactobacillus* species, primer pairs were designed from the conserved regions of 37 *Lactobacillus* species in the 16S rRNA gene sequence and used as an internal positive control (IPC). All primer pairs were designed using Primer Designer (Scientific and Educational Software, Durham, NC, USA) and synthesized by Bionics Co. Ltd. (Seoul, South Korea).

### Specificity of species-specific primer pairs

PCR assays were performed to confirm the specificity of the designed species-specific primer pairs. The specificity was evaluated using 37 *Lactobacillus* reference strains. PCR products were amplified using the following conditions in a thermocycler (Astec, Fukuoka, Japan): 94 °C for 10 min, followed by 30 cycles of 94 °C for 30 s, 60 °C for 30 s, 72 °C for 30 s, and 72 °C for 5 min. The 25 μL reaction mixtures contained 20 ng of template DNA of a *Lactobacillus* reference strain, 0.5 unit of *Taq* DNA polymerase (TaKaRa BIO Inc., Tokyo, Japan), and species-specific primer pairs. The optimal concentration of each species-specific primer pair obtained from the experiments is shown in Table 1. The amplification products were confirmed by electrophoresis on a 2% agarose gel, and the product bands were visualized under a UV transilluminator (Vilber Lourmat, Marne La Vallee, France).

### Development of PCR assays

In this study, we developed the PCR assays that allows each primer pair to run independently to cover each full assays using one primer pair in each well and 37 wells. The PCR assays were performed on the 7500 Real-Time PCR System (Applied Biosystems, Foster City, CA, USA) using the following conditions: 95 °C for 2 min, followed by 30 cycles of 95 °C for 5 s and 60 °C for 30 s. The melting curve data were generated using 1 cycle of 95 °C for 15 s, 60 °C for 1 min, 95 °C for 30 s, and 60 °C for 15 s. The amplification mixture with a final volume of 20 μL for real-time PCR assays included 2X LeGene SB-Green Real-Time PCR Master Mix (LeGene Biosciences, San Diego, CA, USA), template DNA, and species-specific primer pairs at optimal concentrations shown in Table 1. To evaluate the analytical accuracy of the PCR assays, a standard curve was constructed using serial dilutions (50 to 0.005 ng) of genomic DNA from *Lactobacillus* reference strains in triplicate. The specificities of the species-specific primer pairs were tested using 20 ng of DNA extracted from 37 *Lactobacillus* reference strains. PCR amplifications of IPC were also confirmed with 37 *Lactobacillus* reference strains. The results of the PCR were confirmed using 7500 Software V2.3 (Applied Biosystems).

### Application of the developed PCR assays in probiotic and dairy products

We designed a validation test to detect 37 *Lactobacillus* species with PCR in a single 96 well plate using primer pairs. Each well of a reaction plate contained each primer pair and IPC for the detection of 37 *Lactobacillus* species (Additional file 2: Fig. S1). Briefly, 20 ng of product DNA and 2X Master Mix (LeGene Biosciences) were added to each well of the reaction plate containing species-specific primers. Then, PCR was performed in the 7500 Real-Time PCR system (Applied Biosystems). The real-time PCR conditions were similar to those described in “Development of PCR assays” section. Our method included one primer pair in each well, so 37 wells were used for the full assay of each product sample. Therefore, for all products, including mixed samples, the PCR results determined that the corresponding species was included in the product when amplified in a well containing specific primer pair.

## Supplementary information

**Additional file 1: Table S1.** General genome features of *Lactobacillus* species.

**Additional file 2: Figure S1.** Real-time PCR 96-well plate layout for validation of probiotic products. P: Internal positive control, N: no template control, 1: *L. gasseri* specific primer set, 2: *L. rhamnosus* specific primer set, 3: *L. brevis* specific primer set, 4: *L. sakei* specific primer set, 5: *L. johnsonii* specific primer set, 6: *L. jensenii* specific primer set, 7: *L. fermentum* specific primer set, 8: *L. plantarum* specific primer set, 9: *L. paracasei* specific primer set, 10: *L. paraplantarum* specific primer set, 11: *L. casei* specific primer set, 12: *L. curvatus* specific primer set, 13: *L. acidophilus* specific primer set, 14: *L. salivarius* specific primer set, 15: *L. reuteri* specific primer set, 16: *L. coryniformis* specific primer set, 17: *L. farciminis* specific primer set, 18: *L. zymae* specific primer set, 19: *L. pentosus* specific primer set, 20: *L. crustorum* specific primer set, 21: *L. mucosae* specific primer set, 22: *L. buchneri* specific primer set, 23: *L. helveticus* specific primer set, 24: *L. amylovorus* specific primer set, 25: *L. heilongjiangensis* specific primer set, 26: *L. parabuchneri* specific primer set, 27: *L. acidipiscis* specific primer set, 28: *L. sanfranciscensis* specific primer set, 29: *L. ruminis* specific primer set, 30: *L. agilis* specific primer set, 31: *L. delbrueckii* specific primer set, 32: *L. amylophilus* specific primer set, 33: *L. kunkeei* specific primer set, 34: *L. acetotolerans* specific primer set, 35: *L. lindneri* specific primer set, 36: *L. gallinarum* specific primer set, 37: *L. amylolyticus* specific primer set.

## Data Availability

The datasets used and/or analyzed during the current study are available from the corresponding author on reasonable request.

## Abbreviations

*PCR*: Polymerase Chain Reaction
*GRAS*: Generally Recognized As Safe
*16S rRNA*: 16S ribosomal Ribonucleic Acid
*LAB*: Lactic acid bacteria
*DGGE*: Denaturing Gradient Gel Electrophoresis
*IPC*: Internal Positive Control
*MFS*: Major Facilitator Superfamily
*BLAST*: Basic Local Alignment Search Tool
*NCBI*: National Center for Biotechnology Information
*GHKL*: Gyrase, Hsp90, Histidin Kinase, MutL
