# Supplementary material for: Design of PCR assays to specifically detect and identify 37 Lactobacillus species in a single 96 well plate
Source: BMC Microbiol. 2020 Apr 15;20:96. doi: 10.1186/s12866-020-01781-z (PMC7160897; doi:10.1186/s12866-020-01781-z)
Supplement: Supplementary file 2 — Additional file 2: Figure S1. Real-time PCR 96-well plate layout for validation of probiotic products. P: Internal positive control, N: no template control, 1: L. gasseri specific primer set, 2: L. rhamnosus specific primer set, 3: L. brevis specific primer set, 4: L. sakei specific primer set, 5: L. johnsonii specific primer set, 6: L. jensenii specific primer set, 7: L. fermentum specific primer set, 8: L. plantarum specific primer set, 9: L. paracasei specific primer set, 10: L. paraplantarum specific primer set, 11: L. casei specific primer set, 12: L. curvatus specific primer set, 13: L. acidophilus specific primer set, 14: L. salivarius specific primer set, 15: L. reuteri specific primer set, 16: L. coryniformis specific primer set, 17: L. farciminis specific primer set, 18: L. zymae specific primer set, 19: L. pentosus specific primer set, 20: L. crustorum specific primer set, 21: L. mucosae specific primer set, 22: L. buchneri specific primer set, 23: L. helveticus specific primer set, 24: L. amylovorus specific primer set, 25: L. heilongjiangensis specific primer set, 26: L. parabuchneri specific primer set, 27: L. acidipiscis specific primer set, 28: L. sanfranciscensis specific primer set, 29: L. ruminis specific primer set, 30: L. agilis specific primer set, 31: L. delbrueckii specific primer set, 32: L. amylophilus specific primer set, 33: L. kunkeei specific primer set, 34: L. acetotolerans specific primer set, 35: L. lindneri specific primer set, 36: L. gallinarum specific primer set, 37: L. amylolyticus specific primer set. [file 12866_2020_1781_MOESM2_ESM.docx]

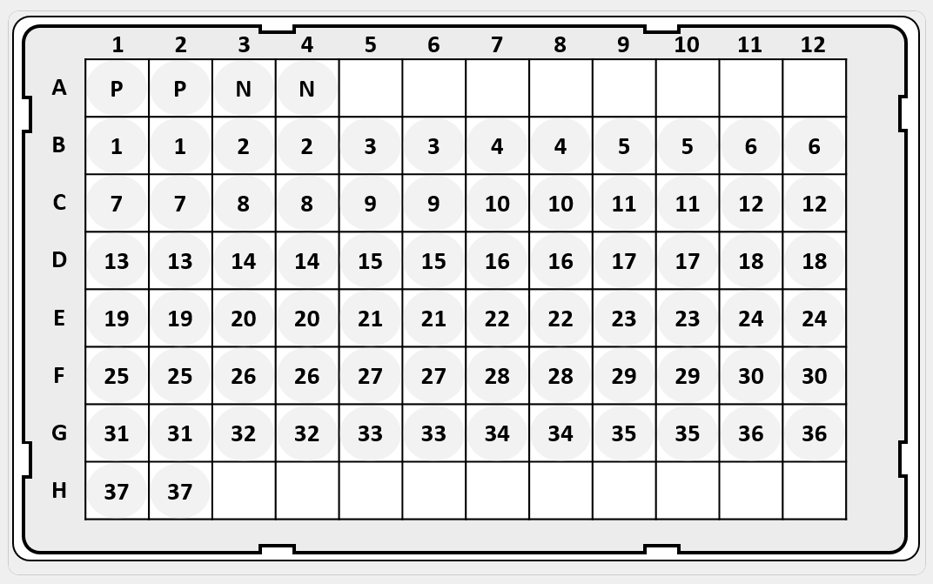


**Fig. S1.** Real-time PCR 96-well plate layout for validation of probiotic products. P: Internal positive control, N: no template control, 1: *L. gasseri* specific primer set, 2: *L. rhamnosus* specific primer set, 3: *L. brevis* specific primer set, 4: *L. sakei* specific primer set, 5: *L. johnsonii* specific primer set, 6: *L. jensenii* specific primer set, 7: *L. fermentum* specific primer set, 8: *L. plantarum* specific primer set, 9: *L. paracasei* specific primer set, 10: *L. paraplantarum* specific primer set, 11: *L. casei* specific primer set, 12: *L. curvatus* specific primer set, 13: *L. acidophilus* specific primer set, 14: *L. salivarius* specific primer set, 15: *L. reuteri* specific primer set, 16: *L. coryniformis* specific primer set, 17: *L. farciminis* specific primer set, 18: *L. zymae* specific primer set, 19: *L. pentosus* specific primer set, 20: *L. crustorum* specific primer set, 21: *L. mucosae* specific primer set, 22: *L. buchneri* specific primer set, 23: *L. helveticus* specific primer set, 24: *L. amylovorus* specific primer set, 25: *L. heilongjiangensis* specific primer set, 26: *L. parabuchneri* specific primer set, 27: *L. acidipiscis* specific primer set, 28: *L. sanfranciscensis* specific primer set, 29: *L. ruminis* specific primer set, 30: *L. agilis* specific primer set, 31: *L. delbrueckii* specific primer set, 32: *L. amylophilus* specific primer set, 33: *L. kunkeei* specific primer set, 34: *L. acetotolerans* specific primer set, 35: *L. lindneri* specific primer set, 36: *L. gallinarum* specific primer set, 37: *L. amylolyticus* specific primer set.
